# Low-temperature catalyst based Hydrothermal liquefaction of harmful Macroalgal blooms, and aqueous phase nutrient recycling by microalgae

**DOI:** 10.1038/s41598-019-47664-w

**Published:** 2019-08-06

**Authors:** Vinod Kumar, Sanjay Kumar, P. K. Chauhan, Monu Verma, Vivekanand Bahuguna, Harish Chandra Joshi, Waseem Ahmad, Poonam Negi, Nishesh Sharma, Bharti Ramola, Indra Rautela, Manisha Nanda, Mikhail S. Vlaskin

**Affiliations:** 1 Department of Chemistry, Uttaranchal University, Dehradun, 248007 India; 2 Department of Life Sciences, Food Technology, Graphic Era Deemed to be University, Dehradun, 248001 India; 3 Faculty of Applied Sciences and Biotechnology, Shoolini University, Solan, HP India; 4 Department of Chemistry, Amity School of Applied Sciences (ASAS), Amity University Haryana, Gurugram-122413, Haryana India; 5 Department of Biotechnology, Uttaranchal University, Dehradun, 248007 India; 6 Department of Biotechnology, Dolphin (PG) Institute of Biomedical and Natural Sciences, Dehradun, 248007 India; 7 Joint Institute for High Temperatures of the Russian Academy of Sciences, 13/2 Izhorskaya St, Moscow, 125412 Russia

**Keywords:** Chemical modification, Chemical modification

## Abstract

The present study investigates the hydrothermal liquefaction (HTL) of harmful green macroalgal blooms at a temperature of 270 °C with, and without a catalyst with a holding time of 45 min. The effect of different catalysts on the HTL product yield was also studied. Two separation methods were used for recovering the biocrude oil yield from the solid phase. On comparision with other catalyst, Na_2_CO_3_ was found to produce higher yiled of bio-oil. The total bio-oil yield was 20.10% with Na_2_CO_3_, 18.74% with TiO_2_, 17.37% with CaO, and 14.6% without a catalyst. The aqueous phase was analyzed for TOC, COD, TN, and TP to determine the nutrient enrichment of water phase for microalgae cultivation. Growth of four microalgae strains *viz*., *Chlorella Minutissima*, *Chlorella sorokiniana* UUIND6, *Chlorella singularis* UUIND5 and *Scenedesmus abundans* in the aqueous phase were studied, and compared with a standard growth medium. The results indicate that harmful macroalgal blooms are a suitable feedstock for HTL, and its aqueous phase offers a promising nutrient source for microalgae.

## Introduction

The appearance of a dense mat of macroalgae on water bodies is a widespread phenomenon. An algal bloom is a result of accumulating algal biomass in the slow-moving lake, pond or river. Macroalgae blooms are largely filamentous, unattached forms, and are mainly green algal species found in nutrient-rich, and temperate waters. Increase of nitrogen and other micropollutants into water bodies are linked to increases in macroalgal blooms worldwide^1^.

Accumulation of macroalgae in rivers and ponds leads to microbial decomposition that may reduce dissolved oxygen in the water bodies due to algal respiration^2^. Low dissolved oxygen content in water bodies leads to a change in biodiversity and species composition^3^. Macroalgal blooms lead to a decline in the growth of non-blooming algae and also affects the diversity of plankton and zooplankton in water bodies^4^. These algal blooms affect the ecosystem by changing the quality of water, light penetration, and alteration of the food chain, and food web^5^. Various initiatives have been undertaken to deracinate the problem related to algal blooms contaminate freshwater as well as marine water^5^. Many studies have been reported on producing biofuels from microalgae^6^. However, a limited number of studies are related to the use of macroalgae for biofuel production. Toxic algal blooms can be used as a cheap raw material for microalgae cultivation because its density has extensively increased worldwide in recent years due to wastewater being discharged into water bodies^7^.

Hydrothermal liquefaction (HTL) is a process in which wet algal feedstocks at 200–400 °C temperature and 10–15 MPa pressure gets converted into four products (1) Bio-crude oil (2) Gas (3) Solid phase and (4) Aqueous phase rich in organics and nutrients^8^. During the HTL process, along with lipids, proteins and carbohydrates also get converted into biocrude oil. Therefore, the yield of oil is higher using HTL^8,9^. Thus, HTL is well suited to a variety of biomass, including bacteria, wastewater sludge, and algal biomass, which is fast-growing but have low-lipid contents. This process provides the energetic advantages by the use of wet algal biomass and the efficient separation of products over alternative techniques such as lipid isolation and transesterification, pyrolysis etc^9^. HTL is ideal for conversion of high-moisture biomass into biocrude oil because water acts as a reaction medium and thus avoids the costly phase of biomass drying^9^.

The use of a catalyst was found more to be suitable for HTL of macroalgae as compared to microalgae because it increases the conversion of carbohydrates into biocrude oil^10^. Previous studies have shown that most of the catalysts lead to a significant increase in biocrude oil. HTL process will not be economically viable if these catalysts cannot be recycled properly^11,12^. Na_2_CO_3_ increases the HTL biocrude oil yield of terrestrial plants, and microalgae by promoting hydrolysis^12^.

Quality of the biocrude oil is dependent on the properties of the algal biomass. High carbon, hydrogen content and low ash, nitrogen, sulfur, and oxygen content containing biomass are considered as ideal for HTL^13^. HTL bio-crude ids dark in color, highly viscous liquid which is 10–10,000 times higher than that of conventional fuel and have a smoke-like smell^14^. High nitrogen content of algal biomass results in higher nitrogen content in HTL biocrude oil. This causes emission of toxic NOx which can be removed by the refining process. The high carbon content of algal biomass resulted in high biocrude yield by HTL process^10^. Composition of biocrude oil is mainly carbon content (71–73%), hydrogen content (7–8%), oxygen content (10–11%), nitrogen content (6–7%) and sulfur content (0–1%) which leads to less greenhouse gas emissions as compared to conventional biofuel and bioethanol^10^. Biocrude can be upgraded for the production of gasoline, jet fuel, and other fuel by using a suitable catalyst which can remove oxygen, nitrogen and double bonds^15^. However, HTL shows a negative energy balance, which is the principal disadvantage of this process. Fast heating and cooling also increase the yield of biocrude oil because of better conversion of protein, lipid, and carbohydrates into liquid fuel rather than solid or gaseous fuel^10^.

According to the energy-efficiency ratio, the use of HTL aqueous phase for microalgae cultivation shows a positive energy balance for biofuel production^16^. Recycling nutrients from wastewater could potentially fulfil the nutrients requirement for microalgae cultivation and scope to integrate the biofuel production and wastewater treatment^17^. Post-hydrothermal liquefaction aqueous phase can accumulate approximately 80% of nutrients and some organics, this provides an excellent opportunity for nutrient and carbon recycling^8^. The nutrients and carbon recycling have been investigated in some recent studies using aqueous phase of HTL^18^. These studies show that nutrients and carbon in the aqueous phase from hydrothermal liquefaction can be used for microalgae cultivation at different dilution factors (50–500 times)^19^. Several studies have been reported in the literature on nutrient cycling of HTL wastewater for microalgae cultivation, but the effect of a different catalyst on the growth of microalgae has not been reported yet. In a study, Jain *et al*.^7^ reported that freshwater toxic algal blooms are a promising feedstock for microalgae cultivation.

**Figure 1:**
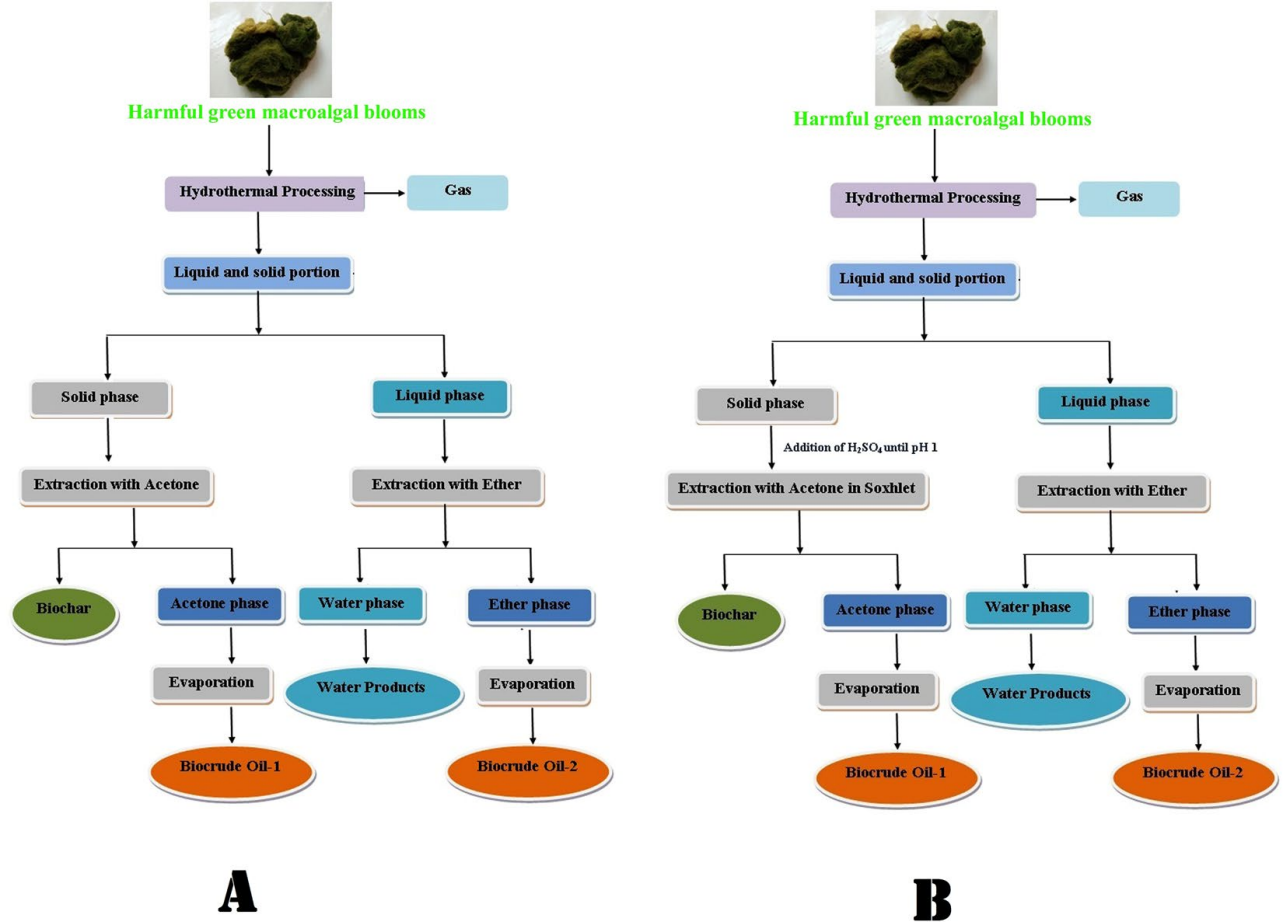
(**A**) Simple separation and extraction procedure. (**B**) Soxhlet based separation and extraction procedure.

The present study investigates explicitly a novel integrated method of using harmful algal blooms as biomass for energy production that synergistically combines algal blooms biofuel production using the HTL process as given in Fig. 1. This study aims to experimentally confirm the feasibility of the harmful green algal blooms for biocrude production completed in four steps. Four steps includes (1) utilization of harmful macroalgal blooms for the HTL process. (2) Increase the yield of biocrude oil using different separation methods. (3) Study the effect of different catalysts on biocrude oil yield. (4) Use of aqueous phase of HTL processes with a catalyst for the cultivation of microalgae.

## Results

### Analysis of macroalgal blooms

The proximate analyses of macroalgal blooms (Supplementary Figs 1–3) are respectively listed in Table 1, 87.23% moisture, 62.01% volatile substantial, 19.02% ash content and 31.19% C, 8.42% H, 4.22% N and 54.61% O. FTIR spectrum reflects (Supplementary Fig. 4) three central regions, lipid band (around 1630 cm^−1^), amide band (1401 cm^−1^) and the carbohydrate region (1100–874 cm^−1^).

**Table 1:** Proximate analysis of harmful algal blooms.

Proximate analysis %	
Moisture (wet biomass)	87.23 ± 0.1
Volatile solid	62.01 ± 0.2
Ash	19.02 ± 0.1
**Elemental analysis %**	
C	31.19 ± 0.3
H	8.42 ± 0.1
N	4.22 ± 0.2
O	54.61 ± 0.2
S	1.56 ± 0.1
HHV	9.45 ± 0.3 MJ kg^−1^

### Product yields by HTL

In this study yields of bio-crude oil by the two separation methods were investigated. In the first separation method (without soxhlet), 10.03% of biocrude oil was obtained. The results indicated that oil yield was high (14.06%) in second separation method (with soxhlet). The Soxhlet extraction method was selected and then further evaluated for their bio-crude oil yield with different catalysts for 45 min at 270 °C as given in Fig. 1. The total bio-oil was 20.10% with Na_2_CO_3_, 18.74% with TiO_2_, 17.37% with CaO, and 14.6% without a catalyst. The biochar yield was 30.12%, 30.05%, 32.09% and 35.84% for Na_2_CO_3,_ TiO_2,_ CaO, and without catalyst respectively. The total biocrude oil yield was measured by the mixing of bio-oil-1 and bio-oil-2. The significant portion of biocrude oil one was obtained from the acetone extraction of the solid phase.

### Analysis of the biocrude oil obtained by HTL

The biocrude oil obtained from the macroalgal blooms have been analyzed by Gas Chromatography (GC), and NIST library was used for the identification of compounds (Table 2). Nine main compounds were identified based on retention area % >1 during direct HTL without catalysts at 270 °C and 45 min of reaction time. The compounds such as amides derivatives, palmitic acid, phenolic compounds, and ketones derivatives, alkanes and alkenes derivatives and some furans were considered as central components of biocrude oil obtained by HTL of algal biomass^20^. Most of the components identified in biocrude oil extracted using macroalgal blooms were somewhat similar to those obtained from microalgae. However, differences in some of the compounds (Pyrrolo [1,2-a]pyrazine-1,4-dione hexahydro-3-(2-methylpropyl and Phytol) were also recorded during the study. This may be due to differences in algal species, composition of the macroalgal blooms and also different GC-MS analysis procedure implemented.

**Table 2:** GC–MS analysis of bio-oil extracted from harmful green macroalgal blooms at 270 °C.

Compounds identified in bio-oil	Area %
3-Penten-2-one, 4-methyl	6.88
2-Pentanone, 3-methylene	5.94
2-Pentadecanone, 6,10,14-trimethyl	16.38
n-Hexadecanoic acid	18.70
Pentadecanoic acid	23.81
Phytol	8.2
Tetradecanoic acid	6.1
Pyrrolo[1,2-a]pyrazine-1,4-dione hexahydro-3-(2-methylpropyl)	4.91
Phenol	3.22

### HHV and ER of algal blooms biocrude oil

The primary elements present in biocrude oils obtained by the catalytic and non-catalytic reaction is given in Table 3. The biocrude oil extracted in the absence of catalyst displayed higher carbon content (70.31%) rather than the macroalgal blooms.

**Table 3:** Properties of biocrude-oil with and without catalyst (wt.% in dry basis).

Reaction	Biocrude oil Yield	C	H	N	S	O*	HHV	Energy recovery	Ash content
No catalyst	14.6 ± 0.4	70.31 ± 0.3	11.03 ± 0.4	7.04 ± 0.3	1.07 ± 0.1	10.24	23.34	36.05	6.1 ± 0.1
Na_2_CO_3_	20.1 ± 0.3	74.83 ± 0.5	12.01 ± 0.3	5.01 ± 0.1	0.93 ± 0.3	7.03	25.59	54.42	4.5 ± 0.1
TiO_2_	18.74 ± 0.4	72.45 ± 0.3	9.03 ± 0.5	8.62 ± 0.1	0.78 ± 0.2	8.01	25.37	50.31	5.7 ± 0.1
CaO	17.37 ± 0.1	71.91 ± 0.3	8.02 ± 0.2	9.66 ± 0.2	1.15 ± 0.2	7.62	23.80	43.74	5.1 ± 0.1

*By difference.

Element enrichment percentage was higher in biocrude oil obtained using Na_2_CO_3_ as a catalyst_._ In the biocrude oil, **t**he nitrogen concentration was less as compared to the green algae. This may be a result of nitrogenous compounds that are obtained in the liquid phase of the HTL process. The amount of nitrogen in the biomass of macroalgal blooms is higher than that of bio-crude oil as a result of generation of nitrogenous compounds during the HTL process in aqueous phase^21^. In contrast to our study, Ross *et al*.^12^ reported that using Na_2_CO_3_ there is an increase in nitrogen content, whereas Jena *et al*.^20^ observed a decrease in nitrogen content of biocrude oil with Na_2_CO_3_ catalyst. The sulfur content was recorded below one wt. % for Na_2_CO_3_ and TiO_2_ based reaction, whereas for petroleum crude oil, it varies between 0 and three wt.%^22^.

HHV of the biocrude oil was obtained with Na_2_CO_3_ (25.59MJ kg^−1^) followed by TiO_2_ (25.37MJ kg^−1^) and CaO (23.80MJ kg^−1^).These values are higher as compared to HHV of macroalgal blooms (9.45MJ kg^−1^).

### Analysis of HTL water phase

Water phase obtained by the HTL of harmful macroalgal blooms had a very foul smell and is dark brown. The pH of catalytic reactions (7.8–8.3) was found to be higher than non-catalytic reaction (7.6) (Table 4). The same pattern of pH was also observed in the aqueous phase of other algal biomass after HTL when Na_2_CO_3_ was used as catalyst^12,22^.

**Table 4:** Chemical characteristics of HTL aqueous phase.

Parameter	Aqueous phase without catalyst	Aqueous phase with CaO	Aqueous phase with TiO_2_	Aqueous phase with Na_2_CO_3_
pH	7.9	7.5	7.8	8.3
COD (mg L^−1^)	25492 ± 05	36025 ± 02	32472 ± 05	40391 ± 04
TN (mg L^−1^)	1491 ± 02	1503 ± 01	1646 ± 03	1805 ± 05
TP (mg L^−1^)	904 ± 04	1034 ± 02	876 ± 03	1023 ± 01
TOC (mg L^−1^)	135 40 ± 01	167 38 ± 05	206 21 ± 02	196 45 ± 04

The high amount of TOC present in the liquid phase of HTL is due to organic matter of feedstock dissolved in water. The nitrogen content in the HTL liquid phase increases in catalyzed reaction because algal proteins get converted into water-soluble amino acids and ammonia^23^.

### Effect of catalysts stress on microalgal growth, biomass and lipid productivity

**Figure 2:**
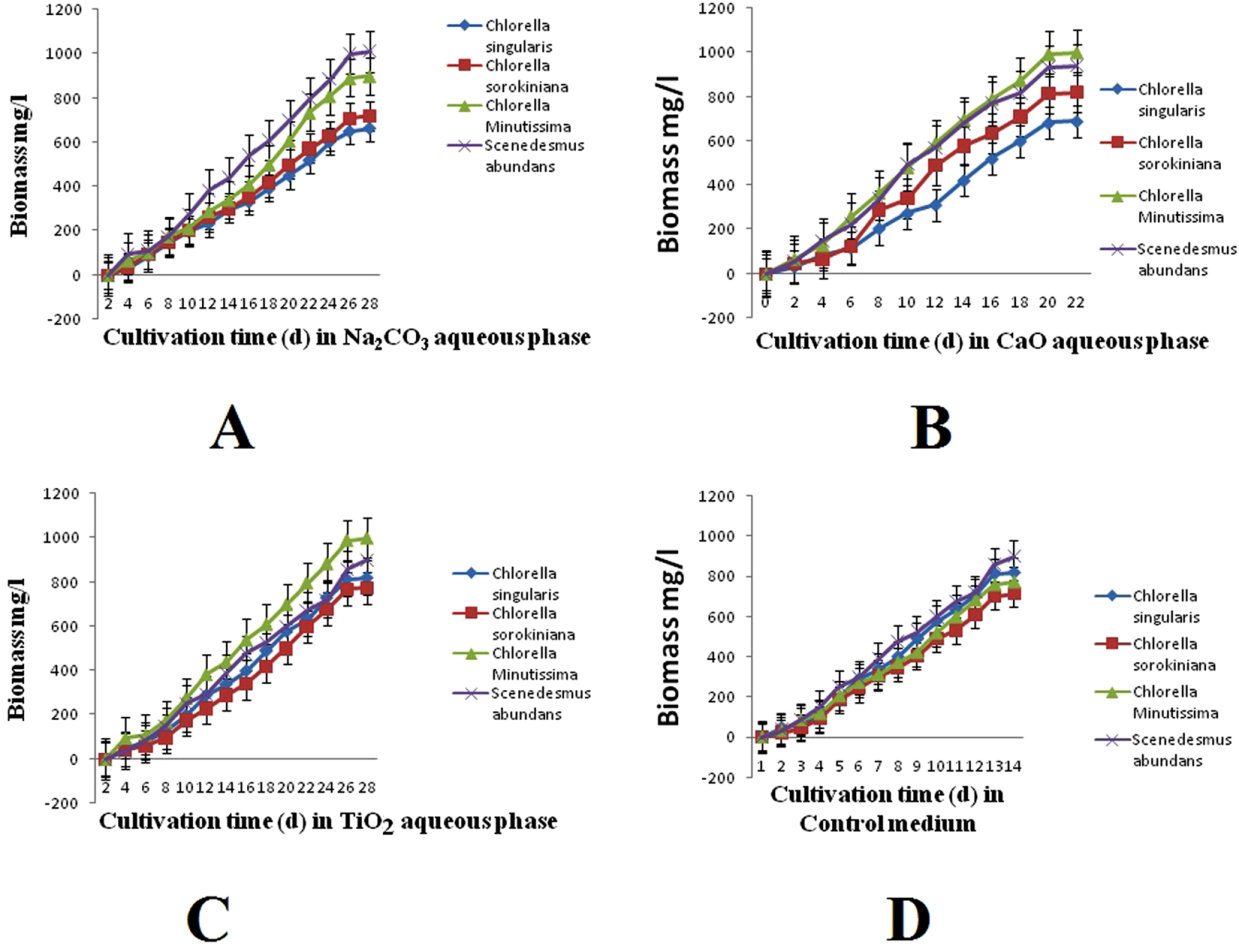
Effect of different catalyst (**A**–**C**) and control medium (**D**) on the growth of microalgae strains.

Microalgal growth in the aqueous phase of each catalyst is different; it may be due to removal and detoxification of catalyst by adsorption of catalysts on to the cell surface or intracellular metabolism of microalgae in response to catalysts. In the aqueous phase of Na_2_CO_3_ and TiO_2_ log phase lasted from day 6 to day 24. While in the aqueous phase of the CaO log phase lasted from day 5 to day 20 (Fig. 2). Overall microalgae, biomass productivity (g/l) after the stationary phase in the control medium (BBM) was found to decrease as compared to the HTL aqueous phase. As Fig. 2 shown that microalgae cultivated in HTL grew with the slow rate but the linear fashion after four days. Each catalyst affects the biomass and lipid productivity differently. Highest lipid yield and productivity of 32 ± 6.1% and 147 ± 1.3 mg L^−1^d^−1^ were recorded in *Scenedesmus abundans*grown in aqueous phase containing catalyst TiO_2_ followed by CaO (*Chorella minutissima* 26.4 ± 1.7%; 157 ± 1.2 mg L^−1^d^−1^), Na_2_CO_3_ (*Scenedesmus abundans* 24.2 ± 2.1%; 110 ± 1.5 mg L^−1^d^−1^), and control (*Chorella minutissima* 22.16 ± 1.6%; 134 ± 2.1 mg L^−1^d^−1^) (Tables 5 and 6).

**Table 5:** Lipid and algal biomass productivity with different aqueous phase at concentration 400× + 1% BBM.

Aqueous phase	*Scenedesmus abundans*		*Chorella minutissima*		*Chlorella sorokiniana*		*Chlorella singularis*	
	Biomass mg/l	Lipid %	Biomass mg/l	Lipid %	Biomass mg/l	Lipid %	Biomass mg/l	Lipid %
CaO	1007 ± 0.5	22.2 ± 0.1	1012 ± 0.1	26.4 ± 1.7	820 ± 0.2	23 ± 4.1	690 ± 0.5	20 ± 4.1
TiO_2_	958 ± 0.1	32 ± 6.1	1003 ± 0.5	28 ± 1.1	768 ± 0.1	26 ± 2.1	835 ± 0.3	23.2 ± 3.4
Na_2_CO_3_	986 ± 0.2	24.2 ± 2.1	900 ± 0.2	21 ± 3.2	732 ± 0.2	22.5 ± 1.1	664 ± 0.1	22 ± 1.1
Control	992 ± 0.4	21 ± 6.1	774 ± 0.2	22.16 ± 1.6	713 ± 0.2	21 ± 3.2	826 ± 0.1	19.2 ± 0.2

**Table 6:** Dry weights of microalgae cultivated in different dilutions of aqueous phase of HTL of harmful algal blooms and in BBM (mg/l).

Microalgae	200×	400×	400× + 1% BBM	600×	BBM
*Scenedesmus abundans*	106 ± 02	759 ± 02	945 ± 02	No growth	900 ± 01
*Chorella minutissima*	204 ± 02	648 ± 01	803 ± 02	107 ± 03	774 ± 01
*Chlorella sorokiniana*	67 ± 01	589 ± 02	741 ± 01	No growth	713 ± 02
*Chlorella singularis*	No growth	461 ± 04	798 ± 03	245 ± 03	820 ± 01

## Discussion

FTIR spectrum of harmful macroalgal blooms reflects three central regions, lipid band (around 1630 cm^−1^), amide band (1401 cm^−1^) and the carbohydrate region (1100–874 cm^−1^). Peaks between 2516–1401 cm^−1^ presents the lipid and phenolic content of algal blooms^24^. Higher phenolic content was reported in marine macroalgae^25^. The sharp peak at 1639 cm^−1^ is C=O amide stretching of proteins present in algae^26^.

In the present study, for the first time harmful macroalgal blooms were used in HTL process for biocrude oil production. High-temperature (300–500 °C with holding time of 30–60 min) based HTL process for the conversion of biomass to bio-crude oil have been reported in previous studies^27,28^. However, the low-temperature (250–290) HTL process is still in the trial stage. In this study maximum, 20.1 ± 0.3% oil was obtained in the presence of catalyst Na_2_CO_3_ at 270 °C for 45 min.

The presence of catalysts showed the increase in oil yield and decrease in the biochar formation^29^. Catalysts increase the quality of biocrude oil by two ways (a) Introduction of catalysts at the time of the HTL process. (b) Upgrade the bio-oil quality after HTL. Catalysts from renewable resources are getting attention because they reduce the production cost of biofuels^30^. In this study, eggshell were used to produced CaO catalyst. Use of Na_2_CO_3_ as a catalyst in HTL leads to increases in the yield of biocrude oil from algal blooms. The bio-oil produced with Na_2_CO_3_ also had high heating value. Yeh *et al*.^31^ reported that Na_2_CO_3_ increases the yield ofbiocrude oil during the HTL of algal biomass containing a high content of carbohydrates by converting it into the oil.

In this study maximum yield of crude oil (20.10%) was reported with catalyst Na_2_CO. The presence of Na_2_CO_3_ catalyst has mainly suited the conversion of carbohydrates into biocrude oil in macroalgae and plant-based biomass^12,23^. However, Na_2_CO_3_ is inimical to the conversion of algal lipids to biocrude oil and not suitable for algal species contain high lipid contents^23^. Shakya *et al*.^22^ reported that higher amount of carbohydrates containing microalgae species led to increasing in bio-oil yield when Na_2_CO_3_ is used as a catalyst. In this study, HHV and ER value of algal blooms biocrude oil with catalyst Na_2_CO_3_ were 25.59MJ/Kg and 27.50% respectively. At similar pressure, Alhassan *et al*.^32^ reported the 21.15 ± 0.82 MJ/kg and ER 41.48% of biocrude oil of *Jatropha curcas* cake under 250 °C temperature. The energy efficiency of HTL biocrude oil depends on the HHV^33^.

Many catalysts such as KOH, NaOH, CH_3_COOH, and H_2_SO_4_ have been used by various researchers in HTL of microalgae, and these catalysts can also be reused as growth media for the growth of microalgae along with aqueous phase^12,21^. Wang *et al*.^27^ reported that by using TiO_2_ in HTL of microalgae at 300 °C leads to the highest bio-oil yield and the maximum liquefaction conversion.

The higher organic carbon content of macroalgae was responsible for the higher yield of bio oil^34^. The crude oil obtained from this study was 14–20% dw which is more similar to as reported in different green macroalgal species, *Enteromorpha prolifera* 23.0%^16^, *Oedogonium* 26.2% and Ulva 18.7% of dry weight^34^.

Algal biomass protein first to gets converted into amino acids and finally into amines and amides by HTL process^12^. Ketones and phenols produced during HTL were obtained from carbohydrates by hydrolysis and dehydration process. The lipids were responsible for the generation of alkenes^16^. Change in nitrogen concentration of aqueous phase of HTL depends on the temperature, type of catalyst, and algae strain^12,22^. High nitrogen content was reported in this study with Na_2_CO_3_ as a catalyst. Similar results were observed by Shakya *et al*.^22^, when Na_2_CO_3_ used as a catalyst in HTL of *Isochrysis* and *Pavlova* at low temperature, as Na_2_CO_3_ increased hydrolysis of protein into water-soluble compounds.

The aqueous phase of HTL had a high amount of soluble organic compound, carbon, and nitrogen. Kumar *et al*.^35^ reported that CaCl_2_ increases microalgal biomass productivity. High lipid content was reported in catalysts containing aqueous phase as compared to control. These results were in congruence with other studies in which an increase in lipid content on exposure to heavy metals was reported^36^. Oxidative stress results in degradation of the photosynthetic machinery, while the protective mechanism of algal cells leads to the accumulation of unsaturated fatty acids^37^.

This experimental work confirmed that harmful macroalgal blooms are a suitable feedstock for HTL and aqueous phase can be reuse as a nutrient source for cultivation of microalgal biomass.

## Material and Methods

### Materials

Microalgae strains *Chorella minutissima (*MCC-27), *Scenedesmus abundans* (NCIM 2897), *Chlorella singulari* UUIND,*and Chlorella sorokiniana* UUIND6 were used in present study. All the chemicals, including TiO_2_ and Na_2_CO_3_ and solvents used, were HPLC grade and acquired from Himedia, India.

### Proximate analysis of green macroalgal blooms

Green macroalgal blooms were locally collected during the winter season (December-February 2017) from water pond, and freshwater river nearby Uttaranchal University, Uttarakhand, India. The ash content of algal blooms biomass was determined according to the NREL Analytical Procedure^38^. Elemental compositions of algal blooms were determined by the elemental analyzer (Thermo Fisher, USA). The proximate analysis of the sample was carried out using the standard methods given by the Association of Official Analytical Chemists (AOAC). FTIR analysis (FTIR 6700, NICOLET) of algal blooms biomass frequency range 4000–450 cm^−1^ were used.

### Experimental procedure of hydrothermal liquefaction (HTL)

The Hydrothermal liquefaction (HTL) of harmful macroalgal blooms was carried out in a 100 ml high-pressure autoclave (Parr reactor) operated in a batch mode. Different types of catalysts in different concentrations were used in HTL process reported in the literature, but 10:1 (feedstock: catalyst) ratio has been reported to give the maximum conversion rate of feedstock to crude oil by HTL^27,39^. The reactor was loaded with wet algal blooms with/without catalyst in 10:1 ratio. Three types of catalysts were used in this study *viz*; TiO_2_, Na_2_CO_3,_and CaO. The CaO catalyst was prepared from eggshells according to the protocol given by Niju *et al*.^40^. The reactor was heated up to 270 °C and pressure 4.5 MPa with He gas, a heating rate of 5 °C/min for holding time of 45 min. After the completion of reaction, the reactor was immediately cooled and opened; gas was vented off, water phase and solid mixture were separated from each other by vacuum filtration. The filtrate was marked as an aqueous phase which consists of dissolved organic compounds. Two separation methods were used for recovering these products.

In the first separation method, the solid phase was treated with acetone three times to recover the oil phase. Acetone was evaporated at 60 °C, and the resulting oil phase was weighed and marked as Biocrude oil 1. The water phase was treated with diethyl ether. Aspirating out upper phase and diethyl ether was evaporated in a rotary evaporator. The total biocrude oil obtained was measured gravimetrically and marked as biocrude oil 2.

In the second separation, Karagoz *et al*.^29^ method were used with some modificationa, briefly the solid phase was acidified to pH 1–2 with H_2_SO_4_ (1.3 M) overnight and dried next day. Biocrude oil from the solid mixture was extracted using soxhlet extraction apparatus with acetone as solvent until the solvent in the thimble becomes colorless. Allowed to stand for three h, upper pahse was separated out. The lower phase (pallets) contain catalyst or residues of catalyst. Acetone was recovered from the upper phase at 60 °C. The extracted oil phase was weighed and marked as biocrude oil-1. Diethyl ether was added to liquid phase and aspirating out upper phase and evaporated diethyl ether in a rotary evaporator and remaining fraction was measured gravimetrically and marked as biocrude oil- 2.

Biocrude oil- 1 was mixed with biocrude oil- 2 for the calculation of %wt of total biocrude oil. Analysis of biocrude samples was doen using an elemental analyzer (Thermo Fisher, USA).

### HHV and energy recovery (ER)

The essential properties of HTL biocrude oil such as biocrude oil yield percentage, HHV, element enrichment %, and ER were calculated using the empirical formulas given below^27,41^.

$$Biocrude\,oil\,(wt \% )=Biocrude\,oil/algal\,biomass\times 100$$

$$HHV\,(MJ\,k{g}^{-1})=0.3383\times C+1.442\times (H-O/8)$$

$$ER\,( \% )=\frac{Yield\,of\,biocrude\,oil \% \times HHV\,biocrude\,oil \% }{HHV\,feedstock}$$

### Aqueous phase water quality analysis

Aqueous phase quality analysis was done to estimate (TN), i.e., Total Nitrogen (NO_3_^−^, NO_2_^−^& NH_4_^+^) and (TP) Total phosphorus (PO_4_^−^). HACH DR 5000 analyzer was used to measure Total Chemical Oxygen (COD) demand. The Total Organic Carbon (TOC) was analyzed by a TOC analyzer (Shimadzu TOC-V).

### Cultivation of microalgae on the aqueous phase

Four microalgal strains* Chlorella Minutissima*, *Chlorella sorokiniana* UUIND6, *Chlorella singularis* UUIND5, and *Scenedesmus abundans* were used in this study.

*Scenedesmus abundans* wasprocured from NCIM, Pune, India. *Chlorella Minutissima was* procured from IARI, New Delhi, India. *Chlorella singularis* UUIND5 and *Chlorella sorokiniana* UUIND6 were earlier isolated by our group.

Microalgal strains were cultivated in 1 L shake flasks containing different dilutions of the aqueous phase of catalytic and non-catalytic reaction and Bold’s Basal Medium (BBM) as a control for 14 days with 16 h light: 8 h dark photoperiod and irradiated with LED tubes (200 μmol m^−2^ s^−1^). BBM was prepared according to the protocol developed by Guarnieri *et al*.^42^.

Three concentrations 200×, 400× and 600× of the aqueous phase of non-catalytic reaction were prepared by the dilution of distilled water (Table 1). The effect on the growth rate of microalgae strains was observed by taking absorbance at 750 nm using UV–Vis spectrophotometer (Shimadzu 133 model no. 1700). Dilution 400× was capable of growing the maximum biomass and was further evaluated for their efficiency in biomass production with 1% BBM and 400× dilution of the aqueous phase of the catalytic reaction.

### Determination of total lipid content (%, w/w) and lipid productivity of the cultivated microalgae

For extraction of lipids, first of all, samples were dried from the % ml culture broth. Further, the microalgal cells were broken down using liquid nitrogen with the help of a mortar and pestle. The fine powder was obtained from which the lipids were extracted using chloroform:methanol (2:1) kept overnight at room temperature, with constant shaking^35^. The extract obtained was treated with 0.034% MgCl_2_, centrifuged at 3500 rpm for 5 min. The supernatant was washed two-three times with 1 ml of 2 N KCl/methanol (4:1 v/v). 5 ml of chloroform/methanol/water (3:48:47, v/v/v) was added to it. The bottom chloroform layer was transferred to a new test tube, and lipids yield was measured gravimetrically. Lipid production and percentage of lipid were calculated by the following equations^35^:

$${\rm{Lipid}}\,{\rm{yield}}\, \% ={\rm{Lipid}}\,{\rm{content}}\,({\rm{g}})/{\rm{Dry}}\,{\rm{algae}}\,{\rm{biomass}}\,({\rm{g}})\times 100$$

$${\rm{Lipid}}\,{\rm{productivity}}={\rm{Biomass}}\,{\rm{productivity}}\times {\rm{Lipid}}\,{\rm{yield}}\,( \% )/100$$

### Statistical analysis

All the experiments were done in triplicates (n = 3) and are presented in mean value ± SD. A GraphPad Prism software (version7:0) with p < 0.05 was used in this study.

## Supplementary information

Supplementary Material
