# Supplementary material for: Low-temperature catalyst based Hydrothermal liquefaction of harmful Macroalgal blooms, and aqueous phase nutrient recycling by microalgae
Source: Sci Rep. 2019 Aug 6;9:11384. doi: 10.1038/s41598-019-47664-w (PMC6684647; doi:10.1038/s41598-019-47664-w)
Supplement: Supplementary file 1 — Supplementary Material [file 41598_2019_47664_MOESM1_ESM.docx]

**Supplementary Material**

**Low-temperature catalyst based Hydrothermal liquefaction of harmful Macroalgal blooms, and aqueous phase nutrient recycling by microalgae**

# Vinod Kumar*^1^, Sanjay Kumar^2^, P K Chauhan^3^, Monu Verma^4^, Vivekanand Bahuguna^5^, Harish Chandra Joshi^1^, Waseem Ahmad^1^, Poonam Negi^1^, Nishesh Sharma^5^, Bharti Ramola^1^, Indra Rautela^5^, Manisha Nanda*^6^, Mikhail S. Vlaskin^7^

^1^Deptt. of Chemistry, Uttaranchal University, Dehradun-248007,India

**^2^**Deptt. of Life Sciences, Food Science, Graphic Era Deemed to be University, Dehradun-248001, India

^3^ Faculty of Applied Sciences and Biotechnology, Shoolini University, Solan, HP, India

^4^Deptt. of Chemistry, Amity University, Gurgaon, Haryana, India

^5^Deptt. of Biotechnology, Uttaranchal University, Dehradun-248007, India

^6^Deptt. of Biotechnology, Dolphin (PG) Institute of Biomedical and Natural Sciences, Dehradun-248007, India

^7^Joint Institute for High Temperatures, 13/2 Izhorskaya St, Moscow, 125412, Russia


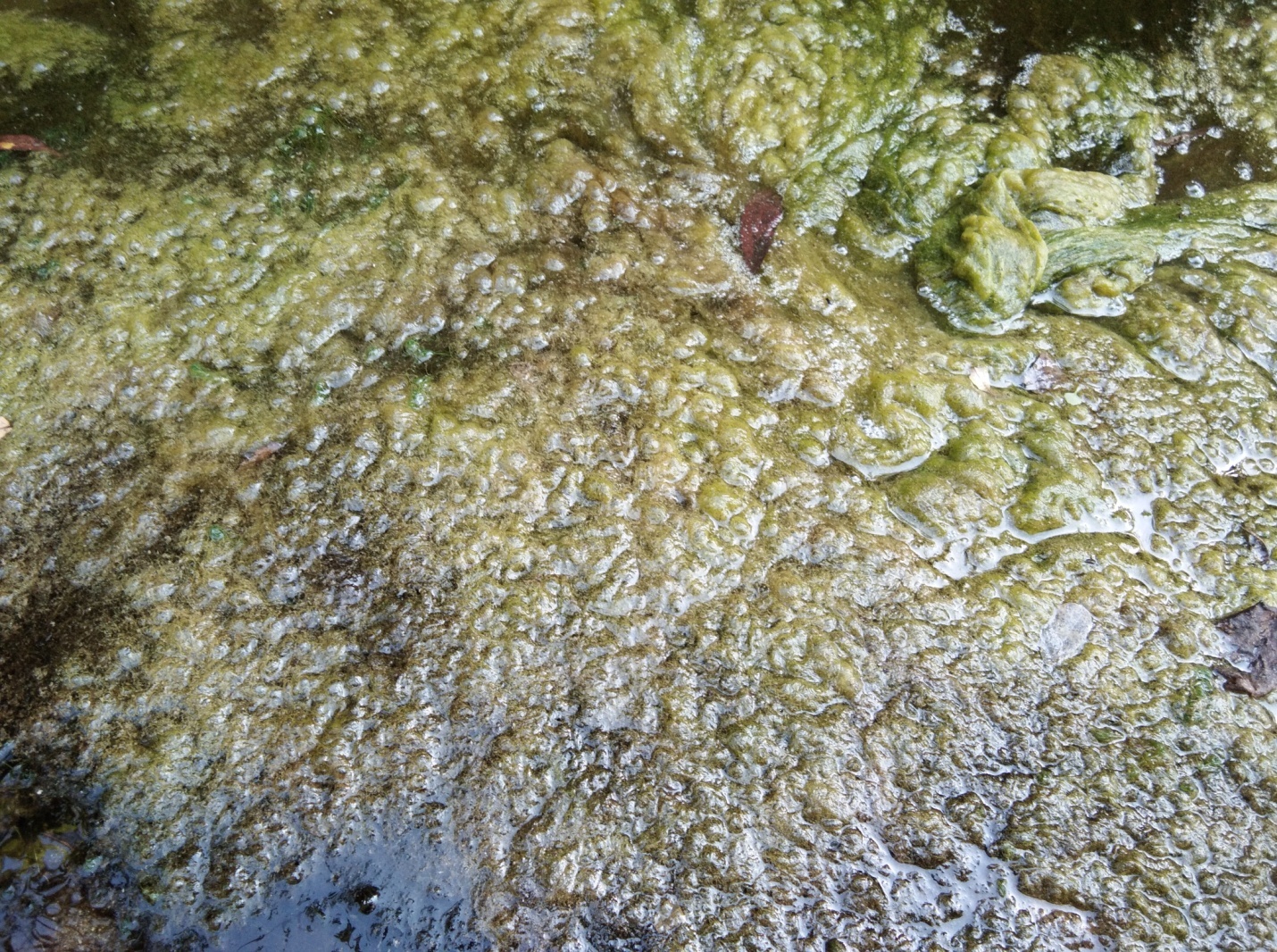


S Fig1: Harmful macroalgal blooms mat in fresh water pond.


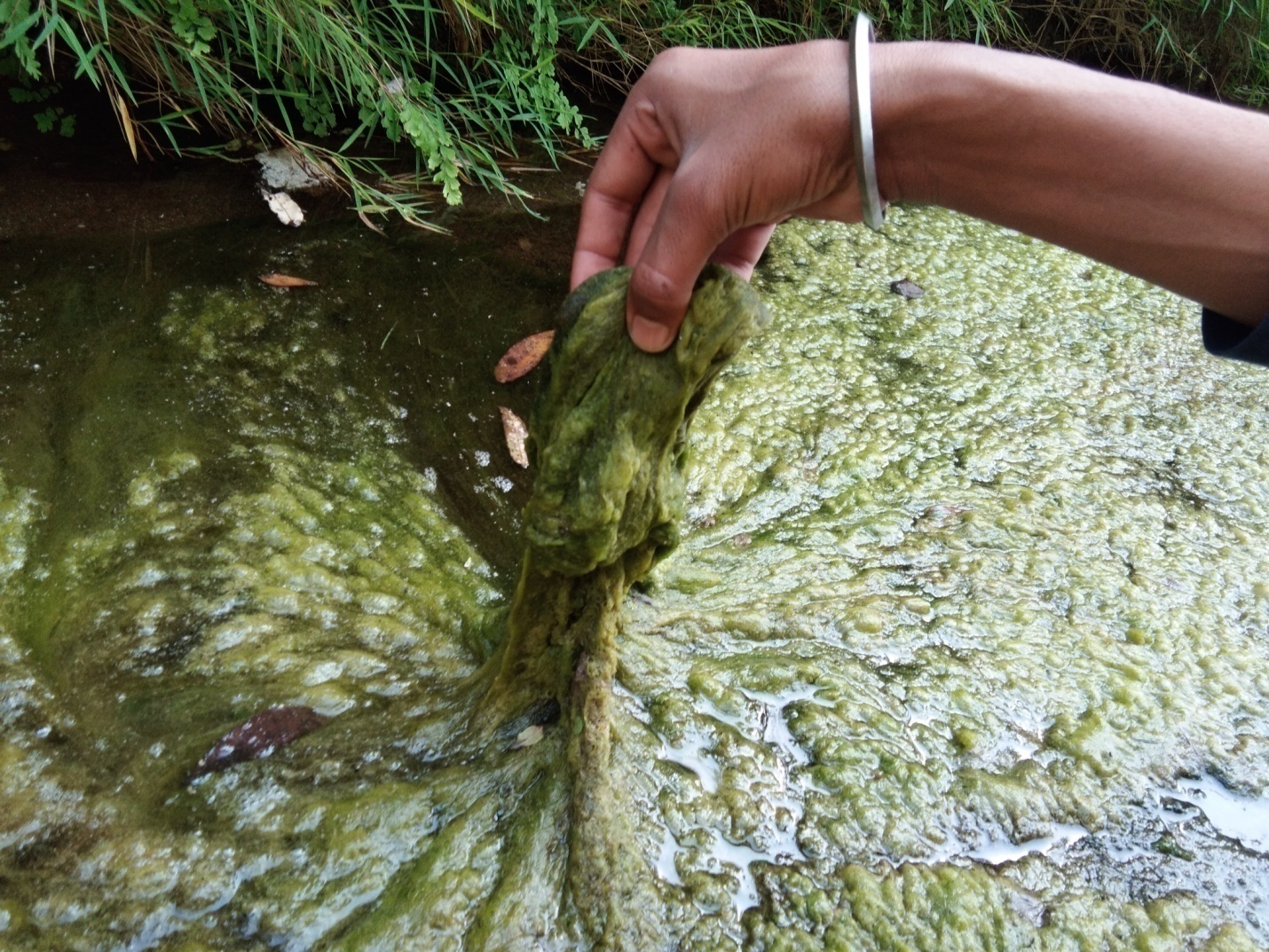


S Fig 2: Collection of Harmful macroalgal blooms


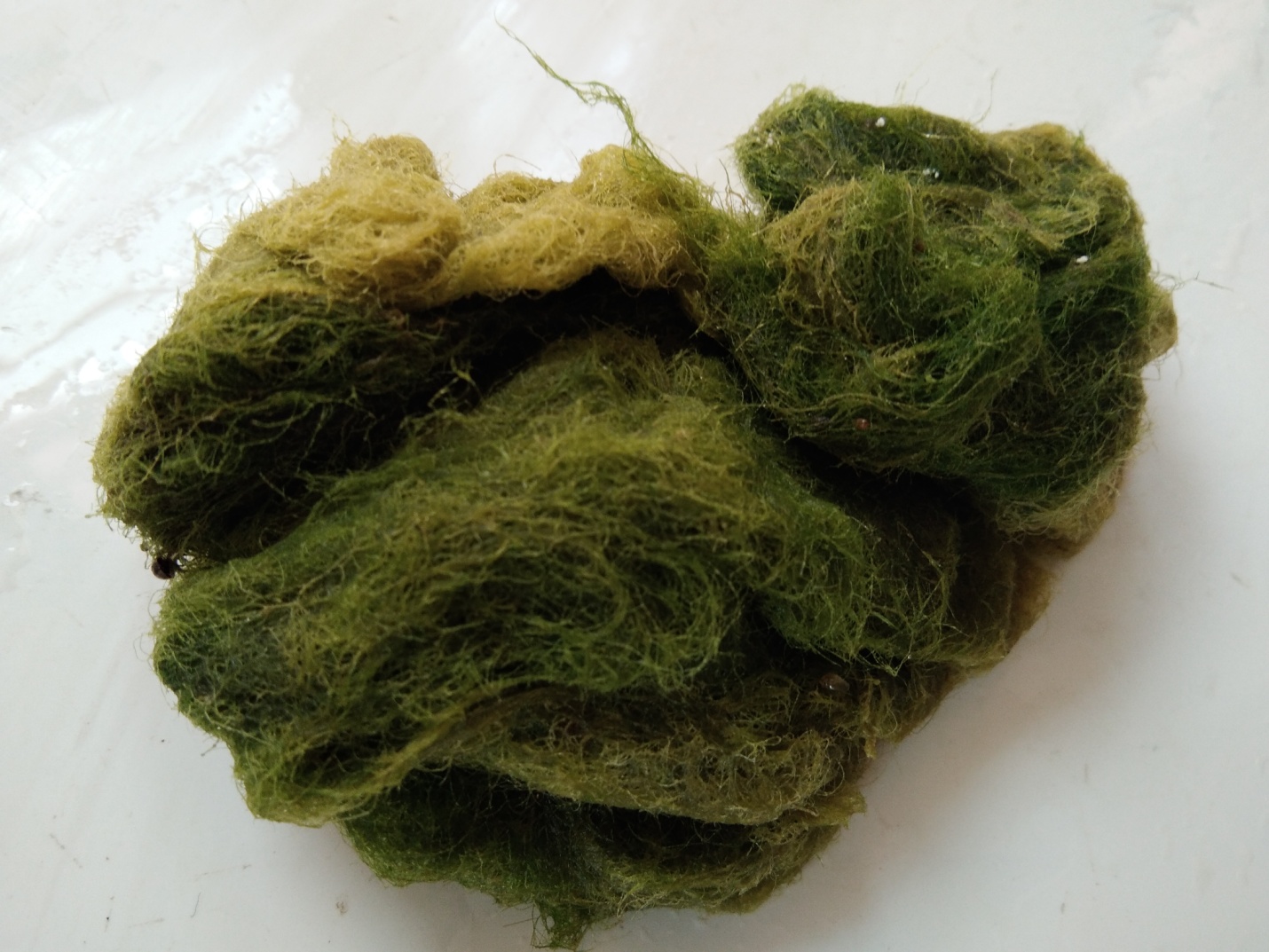
.

S Fig 3: Harmful macroalgal blooms

S Fig 4: FTIR spectrum of harmful macroalgal blooms.
